# The Influence of Contemporary Denture Base Fabrication Methods on Residual Monomer Content, Flexural Strength and Microhardness

**DOI:** 10.3390/ma17051052

**Published:** 2024-02-24

**Authors:** Josip Vuksic, Ana Pilipovic, Tina Poklepovic Pericic, Josip Kranjcic

**Affiliations:** 1 Department of Removable Prosthodontics, University of Zagreb School of Dental Medicine, Gunduliceva 5, 10000 Zagreb, Croatia; jvuksic@sfzg.unizg.hr; 2 Department of Prosthodontics, University Hospital Dubrava, Av. Gojka Šuška 6, 10000 Zagreb, Croatia; 3 University of Zagreb Faculty of Mechanical Engineering and Naval Architecture, Ivana Lučića 5, 10000 Zagreb, Croatia; ana.pilipovic@fsb.unizg.hr; 4 School of Medicine, University of Split, Šoltanska 2, 21000 Split, Croatia; tinapoklepovic@gmail.com; 5 Department of Fixed Prosthodontics, University of Zagreb School of Dental Medicine, Gunduliceva 5, 10000 Zagreb, Croatia

**Keywords:** denture bases, hardness, flexural strength, computer-aided design, computer-aided manufacturing

## Abstract

(1) Background: Digital technologies are available for denture base fabrication, but there is a lack of scientific data on the mechanical and chemical properties of the materials produced in this way. Therefore, the aim of this study was to investigate the residual monomer content, flexural strength and microhardness of denture base materials as well as correlations between investigated parameters. (2) Methods: Seven denture base materials were used: one conventional heat cured polymethyl methacrylate, one polyamide, three subtractive manufactured materials and two additive manufactured materials. High-performance liquid chromatography was used to determine residual monomer content and the test was carried out in accordance with the specification ISO No. 20795-1:2013. Flexural strength was also determined according to the specification ISO No. 20795-1:2013. The Vickers method was used to investigate microhardness. A one-way ANOVA with a Bonferroni post-hoc test was used for the statistical analysis. The Pearson correlation test was used for the correlation analysis. (3) Results: There was a statistically significant difference between the values of residual monomer content of the different denture base materials (*p* < 0.05). Anaxdent pink blank showed the highest value of 3.2% mass fraction, while Polident pink CAD-CAM showed the lowest value of 0.05% mass fraction. The difference between the flexural strength values of the different denture base materials was statistically significant (*p* < 0.05), with values ranging from 62.57 megapascals (MPa) to 103.33 MPa. The difference between the microhardness values for the different denture base materials was statistically significant (*p* < 0.05), and the values obtained ranged from 10.61 to 22.86 Vickers hardness number (VHN). A correlation was found between some results for the material properties investigated (*p* < 0.05). (4) Conclusions: The selection of contemporary digital denture base manufacturing techniques may affect residual monomer content, flexural strength and microhardness but is not the only criterion for achieving favourable properties.

## 1. Introduction

Rehabilitation of edentulous patients with conventional removable dentures is a standard treatment protocol [1,2], and polymethyl methacrylate (PMMA) remains the most commonly used material for the fabrication of denture bases [3,4,5,6,7]. In addition to the well-known analogue techniques for the fabrication of denture bases, contemporary digital technologies are also available today and are used in everyday dental practise. The application of computer-aided design-computer-aided manufacturing technology (CAD-CAM) for complete dentures holds significant potential for patient care, public health, education and research [8]. CAD-CAM technologies include subtractive and additive manufacturing. Digital technologies have been introduced in the manufacture of prosthetic base parts to overcome shortcomings in the properties of prosthetic base materials and to enable faster, more accurate and more cost-effective manufacturing processes. CAD-CAM technology simplifies the laboratory effort [9] and allows for greater automation of procedures, which could result in better denture quality when compared with standard heat-cured PMMA materials [10], less technician time, shorter clinical protocols [8,11,12] and fewer patient visits [9,10].

In addition to procedural advantages [9], it has been hypothesised that CAD-CAM procedures could provide better material properties [10]. The industrial preparation of pre-polymerised discs for subtractive manufacturing is intended to improve material quality by reducing operator dependency [13]. In the industrial polymerisation of pre-polymerised discs for the production of denture bases, polymerisation shrinkage is also no longer an issue [4,12,13,14].

Three-dimensional printing technology is also becoming popular in denture base fabrication and brings additional advantages: it is more economical, there is no wear of rotary instruments, there is less waste of raw materials and it enables the simultaneous manufacture of several products [5,6,15,16,17,18,19]. However, evidence on biocompatibility, mechanical properties, clinical performance and long-term patient follow-up is still lacking [9].

Higher residual monomer concentrations in the denture base material have both mechanical and biological consequences [20]. Residual monomer has a negative effect on the mechanical properties of the denture base material [20,21,22,23,24,25,26,27]. In addition, residual monomer that is leaking in the oral cavity can cause biological reactions in the form of inflammation, irritation and allergic reactions [21,22,24,25,27,28,29]. Residual monomers not only pose a potential risk to the patient but can also pose an occupational risk to the clinician and technician [30]. For this reason, the concentration of residual monomer in the denture base material is one of the most important properties that should be considered.

High flexural strength is required to prevent catastrophic failure of the denture under load [31]. The three-point bending test used to investigate flexural strength simulates the type of load applied to the denture during mastication [26,31,32]. It has been reported that the flexural strength of the denture base material is related to the residual monomer content [10,32].

Microhardness is an important property that is related to the material’s resistance to surface abrasion caused by occlusion and mechanical denture cleaning [23] and to the longevity of the denture [26]. Microhardness is thought to be sensitive to residual monomer content and is a simple way to assess the degree of conversion of the monomer [21,23,33]. There is some evidence of a correlation between microhardness testing and flexural properties [34].

The aim of this study was to investigate the residual monomer content, flexural strength and microhardness of denture base materials fabricated using different manufacturing methods, with a focus on CAD-CAM technology. The aim was also to investigate whether correlations exist between the investigated material properties.

The null hypothesis was that there is no difference in residual monomer content, flexural strength and microhardness between different denture base materials and that there are no correlations between the investigated properties.

## 2. Materials and Methods

The residual monomer content, flexural strength and microhardness of denture base materials were investigated. Seven different denture base materials were used (Table 1). All specimens were prepared according to the manufacturer’s instructions.

Residual monomer

All materials from Table 1 were used in the analysis of residual monomers, with the exception of polyamide, which does not contain methyl methacrylate (MMA) due to its different chemical composition. In accordance with ISO 20795-1:2013 [35], high-performance liquid chromatography (HPLC) was used for the analysis. The specimens were discs with a diameter of 50 mm and a thickness of 3 ± 0.1 mm. All specimens were slightly oversized and were wet-ground with metallographic grinding papers with a grain size of approximately 30 µm (P 500) and 15 µm (P 1200) until the final dimensions were reached. Water was used during the grinding process to avoid any frictional heat that could lead to monomer loss or depolymerisation. To keep the monomer content constant, the specimens were stored in the freezer after preparation until HPLC. For each denture base material, three specimens were prepared and three measurements were performed for each specimen, totalling 54 measurements. The sample size was determined according to ISO 20795-1:2013 [35].

The list of chemicals used in HPLC are shown in Table 2.

Three solutions were prepared with the aforementioned chemicals: solution A, B and C. Solution A was 20 mg L^−1^ mass concentration of hydroquinone in acetone. Solution B was 20 mg L^−1^ mass concentration of hydroquinone in methanol. Solution C was a mixture of one part of solution A and four parts by volume of solution B. 

**Figure 1:**
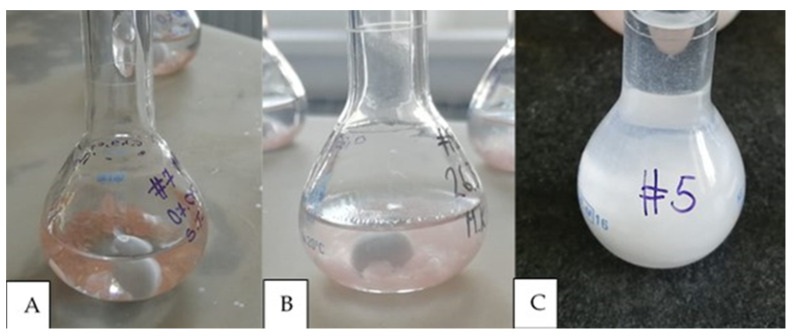
Sample placed in solution A (**A**), after 72 h of dissolving in solution A (**B**), and sample in solution B (**C**).

Prior to chromatography, extraction of the monomer was performed (Figure 1). First, each specimen disc was broken into small pieces, which were additionally ground using a universal laboratory mill with water cooling (M 20, IKA, Aachen, Germany). Grinding was carried out in 3 s pulses with 20 s pauses to avoid frictional heat and monomer losses.

A sample of approximately 650 mg was placed in a 25 mL glass volumetric flask and 10 mL of solution A was added. Each sample was weighed using an analytical balance and the mass was recorded. Acetone in solution A was used to dissolve the sample and hydroquinone in the same solution to avoid the polymerisation of the dissolved residual methyl methacrylate. After 72 h, 2 mL of the sample solution was transferred to a one-mark 10 mL volumetric glass flask, 10 µL of the internal standard was added and solution B was added to a total volume of 10 mL. Solution B consisted of methanol to precipitate the dissolved polymer and hydroquinone to prevent polymerisation of the dissolved methyl methacrylate. To enhance the precipitation of the polymer, the solution was centrifuged for 15 min (EBA-21, Hettich Zentrifugen, Tuttlingen, Germany). The sample solution was additionally filtered through a syringe filter with a pore size of 0.45 µm (Acrodisc, Pall, Ann Arbor, MI, USA) to remove the remaining dissolved macromolecules that could degrade and clog the HPLC columns. 

Immediately after extraction of the monomer, HPLC was performed.

**Figure 2:**
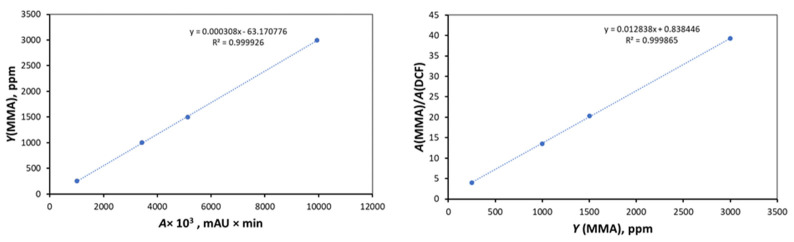
Calibration curve.

Solution C was used to prepare the calibration diagram. It was additionally diluted with ultrapure water prepared with the Direct-Q 3 UV water purification system (Millipore SAS, Molsheim, France). The mixing ratio was solution C/ultrapure water = 66:34. The dilution of solution C was used to improve the separation of the analytes on the chromatograph. Four different concentrations of MMA were used to generate the calibration curve: 0.5, 1, 1.5 and 3 mg/L (Figure 2). Diclofenac at a concentration of 3 mg/L was used for the internal standard.

The concentration of the residual monomer was determined by HPLC with an internal standard. The Shimadzu LC-10 chromatographic system was used (Shimadzu, Kyoto, Japan), which consisted of an SCL-10AVP controller, two LC-10ADvp pumps, a DGU-20AR degasser and an SPD-M10ADvp UV/DAD detector. A Nucleosil C18 RP column (Macherey Nagel, Dueren, Germany) with a length of 250 mm, an inner diameter of 4.6 mm and a pore size of 5 µm was used for the chromatographic separation of the analytes. The mobile phase consisted of two components: the organic component was methanol and the aqueous component was 0.2% formic acid. Each component was pumped individually, and the components were mixed at a ratio of 0.66 parts organic component and 0.34 parts aqueous component with isocratic elution. The total flow rate was 1.0 mL min^−1^ and the volume of the sample solution was 20 µL. Methyl methacrylate was detected at a wavelength of 235 nm and the internal standard at 276 nm.

The concentration of methyl methacrylate was determined using Class VP v6.14 software (Shimadzu, Kyoto, Japan). The mass of MMA in the sample solution, m_MMA_, was calculated using the following Equation:

$$m_{\mathrm{MMA}}=\gamma_{\mathrm{MMA}}\;\times \;V_{e}\;\times \;\frac{V_{p}}{V_{a}}$$

where γ_MMA_ [mg L^−1^] represented MMA concentration, V_e_ [mL] was solution A volume, V_a_ [mL] was a part of the sample solution transferred after dissolving of the sample, and V_p_ [mL] was total volume of the sample solution mixed with solution B and the internal standard. Since V_e_ (10 mL), V_p_ (10 mL) and V_a_ (2 mL) were constant, the Equation was simplified as follows:

m_MMA_ = γ_MMA_ × 0.05

The results of the analysis were calculated according to the following Equation:

$$w=\frac{m_{\mathrm{MMA}}}{m_{\mathrm{SAMPLE}}}\times 100$$

where w [%] represented the mass fraction of MMA in the sample, m_MMA_ [mg] was the mass of MMA in the sample solution and m_SAMPLE_ [mg] was the mass of the sample.

Flexural strength

The flexural strength analysis was performed according to the specifications of ISO 20795-1:2013 [35] and all seven denture base materials from Table 1 were analysed. Five specimen strips were prepared for each denture base material, totalling 35 specimens. They were 64 mm long, 10.0 ± 0.2 mm wide and 3.3 ± 0.2 mm high. All specimen strips were slightly oversized and were wet-ground with metallographic grinding paper with a grain size of approximately 30 µm (P500), 18 µm (P1000) and 15 µm (P1200) until the final dimensions were reached. The prepared specimens were stored in a water bath at a temperature of 37 ± 1 °C for 50 ± 2 h. After removing the specimens from the water bath, flexural testing was immediately performed.

**Figure 3:**
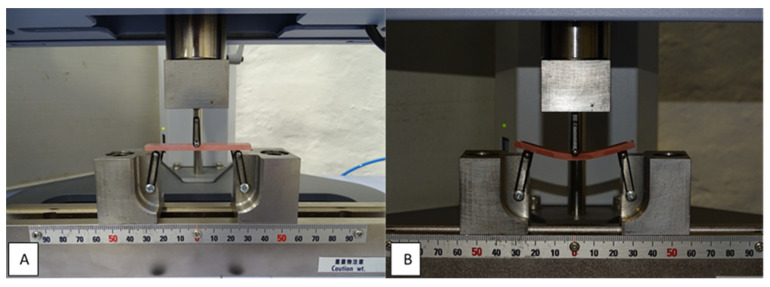
Flexural strength testing: specimen placed in the universal testing machine (**A**), and during the testing (**B**).

Flexural testing was conducted with a universal testing machine (Autograph AGS-X, Shimadzu, Kyoto, Japan). A metal flexural test rig was prepared, consisting of a central loading plunger and two polished cylindrical supports with a diameter of 3.2 mm and a length of 10.5 mm. The supports were arranged parallel and perpendicular to the longitudinal centreline. The distance between the centres of the supports was 50 ± 0.1 mm and the loading plunger was located in the centre between the supports. The displacement rate was 5 mm/min and the test was performed until the specimen broke. The maximum load during the test was recorded (Figure 3).

Flexural strength *σ* [MPa] was calculated using the following Equation:

$$\sigma =\frac{3*F*l}{2*b*h*h}$$

where *F* [N] was the recorded maximum load, *l* [mm] was the distance between the supports, *b* [mm] was the width of the specimen strip and *h* [mm] was the height of the specimen strip.

Microhardness

**Figure 4:**
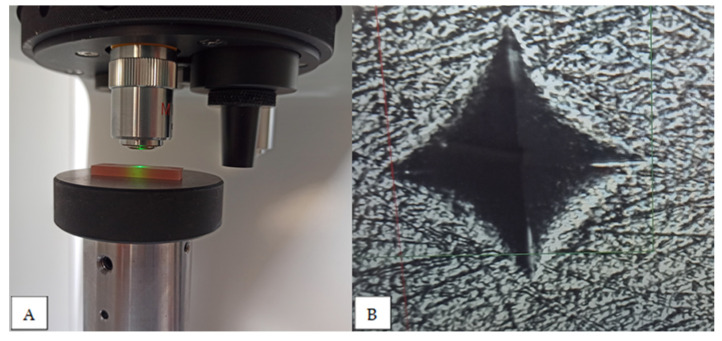
Microhardness testing: specimen placed in the testing machine (**A**), indentation visible on the screen (**B**).

Microhardness analysis was performed using the Vickers method and all materials from Table 1 were analysed. The specimens were 25 × 25 mm plates with a thickness of 3 mm. The specimen plates were wet-ground with P500, P1000 or P4000 metallographic grinding paper and polished with a 0.05 µm aluminium oxide suspension and polishing cloth. The Vickers CSV-10 hardness testing machine (ESI Pruftechnik GmbH, Wendlingen, Germany) was used. The load was 100 g with a dwell time of 15 s. For each denture base material, eight specimens were prepared. Five measurements were performed on each specimen, the Vickers hardness value obtained was recorded and the mean value was calculated for each specimen (Figure 4).

The IBM SPSS software for Windows, version 29.0.1, was used for the statistical analysis. The one-way ANOVA test with a Bonferroni post-hoc test was used for the analysis. Pearson’s correlation coefficient r was used to analyse the correlation between the examined properties. *p* values < 0.05 were considered statistically significant.

## 3. Results

**Figure 5:**
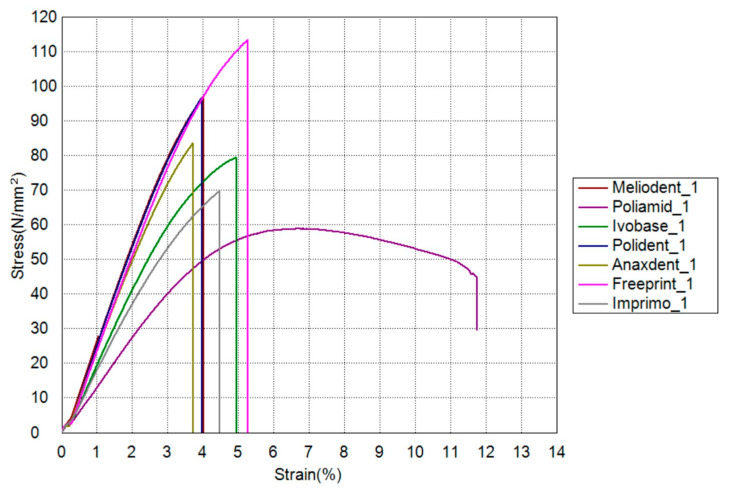
Graph showing flexural stress as a function of strain with average values obtained for each material.

The results of residual monomer content, flexural strength and microhardness are shown in Table 3. A graph presenting flexural stress as a function of strain for flexural strength testing is shown in Figure 5.

The highest value for residual monomer content was obtained for the denture base material Anaxdent pink blank (3.2% mass fraction), while the lowest value was obtained for Polident pink CAD-CAM (0.05% mass fraction). Anaxdent pink blank and Ivobase CAD pink showed statistically significantly higher values for residual monomer than Meliodent heat cure (*p* < 0.001). Polident pink CAD-CAM showed lower values for residual monomer than Meliodent heat cure, but this was not statistically significant (*p* = 0.624). There was no statistically significant difference in the residual monomer content between Meliodent heat cure and additive manufactured materials (Freeprint denture and Imprimo LC denture) (*p* = 1).

For flexural strength, the highest value was obtained for Freeprint denture (103.33 MPa) and the lowest value for Vertex Thermosens (62.57 MPa). Ivobase CAD pink (*p* = 0.037), Imprimo LC denture (*p* < 0.001) and Vertex Thermosens (*p* < 0.001) showed statistically significantly lower values for flexural strength than Meliodent heat cure, while the highest value for Freeprint denture was not statistically significantly different compared to Meliodent heat cure (*p* = 1). There was no statistically significant difference in flexural strength values between three denture base materials for subtractive manufacturing (*p* from 0.055 to 1), while there was a statistically significant difference between two denture base materials for additive manufacturing in terms of flexural strength values (*p* < 0.001). 

In terms of microhardness, the highest value was obtained for Polident pink CAD-CAM (22.86 VHN) and the lowest value for Vertex Thermosens (10.61 VHN). Polident pink CAD-CAM showed statistically significantly different results compared to all other materials (*p* < 0.001). Vertex Thermosens also showed statistically significantly different results compared to all other materials (*p* < 0.001). All materials examined, with the exception of Freeprint denture, showed statistically significantly different results for microhardness compared to Meliodent heat cure (*p* < 0.001). When comparing the microhardness values of all three denture base materials for subtractive manufacturing, there was a statistically significant difference between all materials (*p* < 0.001), and there was a statistically significant difference in microhardness between two denture base materials for additive manufacturing (*p* < 0.001).

The analysis of the correlation is shown in Table 4. When analysing the correlation, a statistically significant negative correlation was found between residual monomer content and the flexural strength value for conventional heat-cured PMMA material (*p* = 0.004). For polyamide denture base material, no statistically significant correlation was found between the investigated properties (*p* = 0.878). For subtractive manufactured materials, a statistically significant negative correlation was found between the residual monomer content and flexural strength (*p* < 0.001) and between the residual monomer content and microhardness (*p* < 0.001). A statistically significant positive correlation between flexural strength and microhardness was also found in the group of subtractive manufactured materials (*p* = 0.001). A statistically significant positive correlation between flexural strength and microhardness was found for additive manufactured materials (*p* = 0.01).

## 4. Discussion

Residual monomer

Since the conventional heat-curing method for MMA polymerisation is widely known, it represents the best reference system for comparison purposes [36], which is why a conventional heat-cured PMMA material (Meliodent heat cure) was included in this study as a control group. The polymerisation reaction and the conversion of MMA is never complete [37], it is unavoidable and zero content cannot be achieved [20,30].

Higher concentrations of residual monomer in denture base materials have both mechanical and biological consequences [20]. Residual monomer acts as a plasticiser by reducing the forces between the chains, so that it negatively influences the mechanical properties of the denture base material [10,20,21,22,23,24,25,26,27,38,39]. Residual monomer that is leaking in the oral cavity can also cause biological reactions in the form of inflammation, irritation and allergic reactions [21,22,24,25,27,28,29]. Various signs and symptoms have been reported in patients as a result of exposure to residual monomer: local chemical irritation, hypersensitivity, mucosal inflammation and ulceration, a burning sensation in the mouth, pain, oedema, swelling and respiratory tract irritation [3,30,40,41,42,43]. Residual monomer is not only a potential risk for the patient but could also represent an occupational risk for the doctor and the technician [30].

To increase the biocompatibility of the denture base material and to achieve optimal material properties, maximum reduction of residual monomer content is desirable [10]. There are a number of factors that can influence the residual monomer content: the mixing ratio of powder and liquid, polymerisation method [30,38], thickness of the denture [44,45], polishing of the surface [3], alternative methods of polymerisation using autoclaves [21], high pressure [32,46] or prolonged curing time [20], post-polymerisation treatments [21,23,40], storage time and storage conditions after fabrication [25,41,45]. It was found that the residual monomer content tends to be lower in dentures that have been used for a longer period of time, but small amounts could still be found in dentures older than 15 years [28].

High pressure and high temperatures are used in the production of pre-polymerised PMMA blocks for subtractive manufacturing, and the process is strictly controlled in the factory [37]. This process promotes the formation of longer polymer chains and should favour a higher degree of monomer conversion and a lower residual monomer content [2,10,14,24,25,46]. As the residual monomer acts as a plasticiser in PMMA material, it is expected that its lower concentration will also improve the mechanical properties [32].

According to ISO 20795-1 [35], various methods can be used to determine the residual monomer content in denture base materials: gas chromatography, high-performance liquid chromatography or any other chromatographic method that gives the same results as the aforementioned methods. Various laboratory techniques for determining residual monomer content can be found in the literature, including UV spectrophotometry [3]. Two different types of residual monomer investigations can also be found in the literature, one is the determination of the amount of residual monomer in the denture base material sample and the other is the determination of the amount of residual monomer released in the water in which the denture base material samples were stored. ISO 20795-1 only proposes analysis of the residual monomer content in the denture base material sample. The requirement of ISO 20795-1 that the upper limit for residual monomer should be 2.2% mass fraction addresses residual monomer content and not residual monomer elution. In our study, we used HPLC to determine the residual monomer content in samples of denture base material as described in ISO 20795-1.

Kedjarune et al. [43] investigated both residual monomer content and residual monomer release in saliva and found that the material with the lowest content has the lowest release, but a higher content does not necessarily mean a higher release.

Ayman et al. [24] showed lower values of residual monomer content in denture base materials for subtractive manufacturing compared to conventional heat-cured PMMA. On the other hand, Steinmassl et al. [10] found no statistical difference in residual monomer release between denture base materials for subtractive manufacturing and conventional PMMA material.

In our study, the results showed that two materials for subtractive manufacturing (Ivobase CAD pink and Anaxdent pink blank) had a statistically significantly higher residual monomer content compared to Meliodent, the standard material for heat-curing PMMA dentures. In addition, the results for Ivobase CAD pink and Anaxdent pink blank did not meet the requirements for residual monomer content specified in ISO 20795-1 (upper limit of 2.2% mass fraction). Ivoclar CAD pink showed the highest value, while the third material for subtractive manufacturing, Polident pink CAD-CAM, showed the lowest value. These differences in the results for the three materials for subtractive manufacturing could indicate that the technology for the production of denture bases is not the only factor relevant for achieving the expected residual monomer content, but that there are also some differences in the composition of the material and probably different industrial procedures for the production of pre-polymerised discs. 

The two materials for additive manufacturing showed lower values compared to Meliodent heat cure, but these were not statistically significantly lower. These results were also well below the upper limit specified in ISO 20795-1. The materials for additive manufacturing differ greatly in composition compared to the heat-cured PMMA materials (the manufacturer stated for Imprimo LC denture that the main component, more than 95%, is bisphenol A polyethylene glycol diether dimethacrylate, and another manufacturer stated for Freeprint denture that the material is MMA-free). There are several explanations for the presence of residual monomer in additive manufactured materials: some amount of MMA could be present in the resin, or it could be a by-product of the photopolymerisation process. It is also possible that a component is present in the material that causes the same reaction as MMA in HPLC on the detector.

Flexural strength

High flexural strength is required to prevent catastrophic failure of the denture under load [4,31]. The three-point bending test used to investigate flexural strength simulates the type of load applied to the denture during mastication [26,31,32,37,47]. Flexural strength is the most commonly used test for dental materials, along with impact strength and microhardness [4,47].

When comparing denture base materials for subtractive manufacturing with conventional PMMA material, recent studies have shown that denture base materials for subtractive manufacturing have statistically significantly higher values for flexural strength [4,12,13,24,31,32,48,49,50,51], while some authors showed similar results for milled denture base materials and conventional PMMA materials [2,4,51]. The results of other authors showed statistically significantly lower measured values for subtractive manufactured denture bases [13,51].

When comparing the flexural strength of additive manufactured materials with conventional heat-cured PMMA material, additive manufactured materials showed statistically significantly lower flexural strength values [4,15,47,51,52], while some authors showed similar results for heat-cured PMMA material [4,53] or even statistically significantly higher values [4].

When comparing subtractive with additive manufactured materials, subtractive manufactured materials showed better results [1,6].

In our study, both subtractive and additive manufactured materials showed statistically significantly lower or similar flexural strength values compared to conventional heat-cured PMMA material. When comparing subtractive manufactured materials with additive manufactured materials, the subtractive manufactured materials showed statistically significantly lower, similar or even higher values. All materials, with the exception of Vertex thermosens, met the criterion of a minimum value of 65 MPa for flexural strength proposed by ISO 20795-1 [35]. It can be concluded that the flexural strength value depends on the specific choice of material and not on the choice of manufacturing process.

The chemical composition of additive manufactured materials is not yet fully provided by the manufacturers, and it seems that the chemical composition of resins for additive manufacturing differs significantly [11,18], so the comparison of different studies could be considered difficult [54].

In the production of pre-polymerised blocks for subtractive manufacturing, the details of the production process are trade secrets [38], but the observed differences in the mechanical properties of the milled denture base materials could also indicate different industrial procedures [32,55].

In order to take full advantage of digital denture manufacturing, it is recommended to further improve resins for additive manufacturing by changing the composition and reinforcement and to optimise processing techniques [2,17,37]. The addition of nanoparticles and nanocomposites [11,18,56,57], build-up orientation, polymerisation technique of the 3D printer, post-curing process, and the number and thickness of the layers can influence the mechanical properties of additive manufactured denture base materials [1,17,47,52,58,59].

Microhardness

Microhardness is an important property of denture base materials that indicates the resistance of the material to surface wear [5,21], which means that loss of smoothness is avoided, and plaque retention and pigmentation are reduced, resulting in a longer useful life of the denture [23]. Microhardness is a measure of resistance to local plastic deformation caused by mechanical indentation or abrasion [1,51]. It is one of the most frequently performed tests on materials. Several different methods are used: Vickers, Brinell and Knoop. The Vickers method is considered a valid tool for microhardness testing [21,23] and is most commonly used for microhardness testing of denture base materials. However, the Vickers method also has some limitations: measurements can be limited by the resolution of the optical system, the operator’s perception and the elastic recovery of the material [23].

Regarding microhardness, recent studies for subtractive manufactured denture base materials found results similar to conventional PMMA materials [2,13], while several authors reported higher [24,32,60] or even lower values of microhardness [12]. For additive manufactured materials, data from recent studies generally showed the lowest values for microhardness compared to conventional PMMA materials [5,18,47,51,57].

In this study, when comparing three subtractive manufactured materials with conventional heat-cured PMMA, one material showed statistically significantly higher values, while the other two materials showed statistically significantly lower values. For the additive manufactured materials, one material showed a statistically significantly lower value, while the other material showed similar values when compared to the conventional heat-cured PMMA material. All of these results are consistent with the findings of previous studies and may indicate that the choice of manufacturing process alone is not the only criterion for achieving the expected microhardness values.

It is assumed that microhardness is sensitive to residual monomer content and is a simple way to evaluate the degree of conversion of the monomer [21,23,26,32,33,34]. The hardness values are directly proportional to the amount of residual monomer [23]. Similarly, flexural strength is also proposed as a simple way to indicate the conversion of the monomer, as it is also sensitive to residual monomer content [32,37,55]. Lee et al. [34] investigated the correlation between the different mechanical properties and showed a high positive correlation between the microhardness test and flexural properties.

Our study also investigated the correlation between the properties of the materials. Our results are partly consistent with previous studies [23,34]. A statistically significant positive correlation between microhardness and flexural strength was found for additive and subtractive manufactured materials, but no statistically significant correlation was found for conventionally heat-cured and polyamide materials. As there is no statistically significant correlation between residual monomer content and other properties investigated for additively manufactured materials, the above suggestions (by other authors) for using microhardness and flexural strength values to determine the monomer are not considered.

## 5. Conclusions

The choice of manufacturing process is not a suitable criterion for achieving desirable values of residual monomer content, flexural strength and microhardness. According to results from this study, it can be concluded that differences between investigated parameters exist, and therefore the null hypothesis is rejected. 

The values for residual monomer content are different for the materials tested. The highest values are found in the group of subtractive manufactured materials, but at the same time, the lowest value was also found in the same group of materials (Polident pink material).

The highest value of flexural strength was found in the group of additive manufactured materials, followed by heat-cured PMMA material and a material from the subtractive manufactured group of materials.

The microhardness values differed between the materials tested, even between materials in the same material group (additive and subtractive manufactured materials).

The lowest values for flexural strength and microhardness are obtained for the material Vertex thermosens.

The values of residual monomer influence flexural strength in a group of subtractive manufactured materials (higher residual monomer with lower values for microhardness and flexural strength) and for conventionally heat-cured PMMA (higher residual monomer with lower values for flexural strength).

## Figures and Tables

**Table 1:** List of the materials used in the study.

Name of the Material	Manufacturer	Description and Purpose of the Material
Meliodent heat cure	Kulzer, Hanau, Germany	Denture base material, PMMA, heat cured
Vertex Thermosens	Vetex Dental, Soesterberg, The Netherlands	Denture base material, polyamide, injection technique
Ivobase CAD pink V	Ivoclar Vivadent, Schaan, Liechtenstein	CAD-CAM denture base material, subtractive manufacturing
Polident pink CAD-CAM disc basic	Polident d.o.o., Volčja draga, Slovenia	CAD-CAM denture base material, subtractive manufacturing
Anaxdent pink blank U medium pink	Anaxdent GmbH, Stuttgart, Germany	CAD-CAM denture base material, subtractive manufacturing
Freeprint denture	Detax, Ettlingen, Germany	CAD-CAM denture base material, additive manufacturing
Imprimo LC denture	Scheu, Iserlohn, Germany	CAD-CAM denture base material, additive manufacturing

CAD-CAM: computer-aided design-computer-aided manufacturing, PMMA: polymethyl methacrylate.

**Table 2:** List of chemicals used in residual monomer investigation.

Chemical Name	Manufacturer	Purity
Acetone	Acros Organics, Geel, Belgium	99.8%
Diclofenac sodium	Sigma Aldrich, St. Louis, MO, USA	≥98%
Hydroquinone	Fluka, Gillingham, UK	≥99%
Methanol	J.T. Baker, Phillipsburg, NJ, USA	≥99.9%
Methil methacrylate, stabilized	Acros Organics, Geel, Belgium	≥99%
Formic acid	Fischer Scientific, Waltham, MA, USA	≥99%

**Table 3:** Residual monomer, flexural strength and microhardness results.

	Residual Monomer [% Mass Fraction]		Flexural Strength [MPa]		Microhardness [VHN]	
	Mean	SD	Mean	SD	Mean	SD
1. Meliodent Heat Cure	0.53 ^3,5^	0.07	97.06 ^2,3,7^	6.25	20.58 ^2,3,4,5,7^	0.52
2. Vertex Thermosens			62.57 ^1,4,5,6^	5.69	10.61 ^1,3,4,5,6,7^	0.24
3. Ivobase CAD Pink	3.05 ^1,4,6,7^	0.58	79.06 ^1,6^	4.65	17.23 ^1,2,4,5,6^	0.99
4. Polident Pink CAD-CAM	0.05 ^3,5^	0.03	96.27 ^2,7^	5.81	22.86 ^1,2,3,4,6,7^	0.72
5. Anaxdent Pink Blank	3.20 ^1,4,6,7^	1.14	83.31 ^2,6^	3.21	18.83 ^1,2,3,4,6,7^	0.48
6. Freeprint Denture	0.36 ^3,5^	0.16	103.33 ^2,3,5,7^	16.71	21.30 ^2,3,4,5,7^	0.45
7. Imprimo LC Denture	0.34 ^3,5^	0.13	69.75 ^1,4,6^	7.63	16.55 ^1,2,4,5,6^	0.81

Mpa = megapascal, VHN = Vickers hardness number, SD = standard deviation. Superscripted numbers indicate a statistically significant difference between materials, *p* < 0.05.

**Table 4:** The results for correlation analysis.

		Microhardness (VHN)	Residual Monomer (% Mass Fraction)	Flexural Strength (Mpa)
**MELIODENT**				
MICROHARDNESS (VHN)	Pearson Correlation	1.000	0.655	−0.822
	P		0.078	0.088
RESIDUAL MONOMER (% mass fraction)	Pearson Correlation	0.655	1.000	−0.976
	P	0.078		0.004 *
FLEXURAL STRENGTH (MPa)	Pearson Correlation	−0.822	−0.976	1.000
	P	0.088	0.004 *	
**VERTEX THERMOSENS**				
MICROHARDNESS (VHN)	Pearson Correlation	1.000	/	−0.096
	P		/	0.878
RESIDUAL MONOMER (% mass fraction)	Pearson Correlation	/	/	/
	P	/	/	/
FLEXURAL STRENGTH (MPa)	Pearson Correlation	−0.096	/	1.000
	P	0.878	/	
**SUBTRACTIVE MANUFACTURED MATERIALS**				
MICROHARDNESS (VHN)	Pearson Correlation	1	−0.815	0.826
	P		0.000 *	0.000 *
RESIDUAL MONOMER (% mass fraction)	Pearson Correlation	−0.815	1	−0.756
	P	0.000 *		0.001 *
FLEXURAL STRENGTH (MPa)	Pearson Correlation	0.826	−0.756	1
	P	0.000 *	0.001 *	
**ADDITIVE MANUFACTURED MATERIALS**				
MICROHARDNESS (VHN)	Pearson Correlation	1	0.074	0.765
	P		0.786	0.010 *
RESIDUAL MONOMER (% mass fraction)	Pearson Correlation	0.074	1	0.215
	P	0.786		0.551
FLEXURAL STRENGTH (MPa)	Pearson Correlation	0.765	0.215	1
	P	0.010 *	0.551	

* indicates statistically significant correlation between investigated properties (*p* < 0.05). Mpa = megapascal, VHN = Vickers hardness number.

## Data Availability

Data are contained within the article.
